# Endocytosis of a Functionally Enhanced GFP-Tagged Transferrin Receptor in CHO Cells

**DOI:** 10.1371/journal.pone.0122452

**Published:** 2015-03-24

**Authors:** Qi He, Xiaoxu Sun, Chong Chu, Qing Jiang, Huifen Zhu, Yong He, Tingting Yue, Ruibo Wang, Ping Lei, Guanxin Shen

**Affiliations:** 1 Department of Immunology, Tongji Medical College, Huazhong University of Science and Technology, Wuhan, Hubei, China; 2 Department of Clinical Laboratory, the First Affiliated Hospital of Zhengzhou University, Zhengzhou, Henan, China; 3 Department of Nuclear Medicine, Union Hospital, Tongji Medical College, Huazhong University of Science and Technology, Hubei Province Key Laboratory of Molecular Imaging, Wuhan, Hubei, China; 4 Beijing Pushikang Pharmaceutical Technology Co., Ltd, Beijing, China; University of Birmingham, UNITED KINGDOM

## Abstract

The endocytosis of transferrin receptor (TfR) has served as a model to study the receptor-targeted cargo delivery system for cancer therapy for many years. To accurately evaluate and optically measure this TfR targeting delivery in vitro, a CHO cell line with enhanced green fluorescent protein (EGFP)-tagged human TfR was established. A chimera of the hTfR and EGFP was engineered by fusing EGFP to the amino terminus of hTfR. Data were provided to demonstrate that hTfR-EGFP chimera was predominantly localized on the plasma membrane with some intracellular fluorescent structures on CHO cells and the EGFP moiety did not affect the endocytosis property of hTfR. Receptor internalization occurred similarly to that of HepG2 cells expressing wild-type hTfR. The internalization percentage of this chimeric receptor was about 81±3% of wild type. Time-dependent co-localization of hTfR-EGFP and PE-conjugated anti-hTfR mAb in living cells demonstrated the trafficking of mAb-receptor complexes through the endosomes followed by segregation of part of the mAb and receptor at the late stages of endocytosis. The CHO-hTfR cells preferentially took up anti-hTfR mAb conjugated nanoparticles. This CHO-hTfR cell line makes it feasible for accurate evaluation and visualization of intracellular trafficking of therapeutic agents conjugated with transferrin or Abs targeting the hTfRs.

## Introduction

The transferrin receptor (TfR, CD71) is a membrane-bound protein involved in transferrin (Tf)-mediated iron uptake. It is expressed on rapidly dividing cells like tumor cells or cell lines in culture. In contrast, in nonproliferating cells, expression of TfR is low or frequently undetectable [1]. The high levels of expression of TfRs in cancer cells, their extracellular accessibility, their ability to internalize, and their central role in the pathology of human cancer make TfR an attractive target that can be exploited for the delivery of cytotoxic agents into tumor cells [2]. Targeting the human TfR has been shown to be effective in delivering therapeutic agents, including chemotherapeutic drugs, cytotoxic proteins, and high molecular weight compounds into cells and causing cytotoxic effects including growth inhibition and/or induction of apoptosis in a variety of malignancies in vitro and in vivo including patients [3]. Our laboratory also developed Tf or anti-hTfR Ab conjugated peptide, polylysine, polyetherimide, nanoparticle delivery systems which exhibited both intrinsic cytotoxic activity and the ability to deliver a wide variety of therapeutic agents into cancer cells [4,5]. Now we focused on anti-TfR Ab mediated drug delivery systems, such as mAb-directed HPPS nanoparticles, multivalent antibody-directed PEI and Au nanoparticles.

In our investigation, in order to accurately evaluate the specificity of this TfR-mediated cargo transport in vitro, a pair of cell lines in which one highly expresses TfR, whereas the other expresses no detectable TfR as control, is needed.

Immunocytochemical ananlysis in chemically fixed cells has been largely used to visualize TfR endocytosis. However, it’s much better to track TfR fluorescence in living cells to show the dynamics of cellular distribution of TfR and its ligands. Stoichiometric labeling of the TfR is useful for further investigation of the therapeutic potential of targeting this receptor.

Given all these, we report here the preparation and the characterization of a CHO cell line which expresses the functional hTfR chimera with enhanced green fluorescent protein (EGFP) that is fused to the amino terminus of the receptor. The EGFP-hTfR chimera on CHO cells retains the internalization functionality as wt-hTfR and CHO-hTfR cells provide a good cell model for evaluating the specificity of anti hTfR mAb-directed nanoparticles *in vitro*.

## Materials and Methods

### Cell culture

CHO cells and K562 cells (China Center for Type Culture Collection, Wuhan, PR China) were cultured in RPMI1640 medium (Invitrogen, Carlsbad, CA, USA) supplemented with 10% fetal bovine serum (FBS, Invitrogen) and 100 U/ml ampicillin, 100mg/ml streptomycin. HepG2 cells (China Center for Type Culture Collection, Wuhan, PR China) were cultured in DMEM complete growth medium (Invitrogen) supplemented with 10% FBS and antibiotics. All cells were incubated at 37°C in a humidified atmosphere containing 5% CO_2_.

### Plasmid construction and transfection

An enhanced GFP was attached to the amino terminus of human TfR by standard recombinant techniques. Briefly, total RNA was extracted from TfR highly expressing K562 cells using Trizol (Invitrogen) following manufacturer's instructions. Total RNA was reverse transcribed to cDNA using ACE reverse transcriptase (Toyobo, Osaka, Japan).The full-length cDNA of TfR was achieved by PCR using forward primer 5'-GCTAAGATCT_BglII_ATGATGGATCAAGCT-3' and reverse primer 5'-GTGTGTCGAC_SalI_TTAAAACTCATTGTC-3'. Then the cDNA was ligated with pGEM-T vector (Promega, Madison, USA) and finally subcloned into pEGFP-C1 (BD Biosciences, CA, USA) to yield pEGFP-hTfR which was verified by DNA sequencing (Invitrogen, Shanghai, China) and restriction enzyme digestion.

Linearized pEGFP-hTfR or empty vector pEGFP-C1 were transfected into CHO cells using Lipofectamine 2000 (Invitrogen) according to the manufacturer's instructions. Stable transformants of CHO cells were obtained by selection with G418 (Promega), and then individual clonal lines of CHO expressing bright fluorescence were established by limited dilution and single cell plating. The sorted pool of EGFP-expressing cells was maintained and used for experiments as indicated. pEGFP-hTfR stable transformants are denoted as CHO-hTfR cells and vector (encoding for green fluorescent protein only) stable transformants as CHOvec cells. HepG2 cell line expressing hTfR-wt was set as an hTfR^+^ control and CHO cell line as an EGFP^-^ hTfR^-^ control in the following experiments.

### Western Blotting

2×10^6^ cells were washed in ice-cold PBS twice and lysed on ice by 150 μl RIPA lysis buffer (Beyotime, Shanghai, China) and 1.5 μl 100 mM PMSF (Roche, Basel, Switzerland) for 1 minute. Cell lysates were centrifuged at 12000 rpm for 5 min and the supernatants were divided on 12% polyacrylamide gel. The transferred membrane was immunoblotted with mouse anti-hTfR monoclonal antibody (anti-hTfR mAb, produced by our laboratory [6–8]) overnight at 4°C followed by HRP-labeled goat anti mouse Ab (Southern Biotech, Birmingham, USA) for 1h at 37°C. Proteins were detected using ECL kit (Pierce, Rockford, USA). The β-actin was set as loading control.

### Flow cytometry analysis

2×10^5^ cells were rinsed 3 times with ice-cold PBS supplemented with 1% BSA,1% serum and then incubated with Alexa Fluor 633 conjugated transferrin (Alexa-Tf, 5 μg per tube, Invitrogen, Tf is purified from human serum.) or anti-hTfR mAb for 1 h at 4°C. After 3 times washes, anti-hTfR mAb treated cells were stained with PE-conjugated donkey anti-mouse Ab (Southern Biotech) at 4°C for 30 min. Half of these cells would be incubated at 37°C for another 30 min to allow internalization. Afterward, all cells were stripped with ice-cold medium 1 (150 mM NaCl and 50 mM glycine acid, pH 2.5) for 1 min, followed by two more washes with RPMI1640 plus 10% FBS. The percentage of fluorescent cells and the mean fluorescence intensity (MFI) were measured by flow cytometry (LSRII, BD Biosciences, USA).

### Immunofluorescence staining in fixed cells and Quantitative colocalization analysis

Cells grown on coverslips were washed twice in PBS and then treated with Alexa-Tf or anti-hTfR mAb the same as in *Flow cytometry analysis*. Cells were then washed intensively and mounted in Antifade Mounting Medium (Beyotime). Fluorescent signal was acquired using confocal microscopy (Olympus, Tokyo, Japan)

Quantitative colocalization analysis was performed using Image Pro Plus software (MediaCybernetics, Bethesda, MD) to calculate a number of coefficients to characterize colocalization.

### Live cell fluorescent imaging and Intracellular localization of mAb-hTfR chimera

CHO-hTfR cells were grown in glass chambers (Corning, NY, USA) overnight. For live cell fluorescent imaging, a Zeiss LSM 710 confocal microscope (Zeiss, Oberkochen, Germany) equipped with Zen-2009 software was used to acquire 2-D time-lapse image series. Cells were cultured with anti-hTfR mAb for 1h at 4°C followed by twice rinsing, and then the chamber was mounted onto the microscope stage. Immediately after the supplement of secondary antibody conjugated with PE to the chamber, the image acquisition through the GFP or PE channel was started. Images were acquired continuously at 2-min intervals for 40 min on the microscope stage. To observe the intracellular localization of mAb and hTfR- EGFP chimera, cells were incubated with anti-hTfR mAb for 1h at 4°C. After 3 times washes, anti-hTfR mAb treated cells were stained with AMCA-conjugated goat anti-mouse Ab (ProteinTech Group) at 4°C for 30 min. Then cells were incubated for another 5min or 30min at 37°C to allow internalization. After fixation in 4% paraformaldehyde, a PE anti-mouse LAMP-1 antibody (eBioscience) /Rabbit anti-mouse EEA-1 (PE seccondary labeling) was used to label intracellular lysosome and endosome. Fluoresecent images were captured by cofocal microscopy.

### Internalization assay

To calculate the internalization percentage of anti-hTfR mAb in CHO-hTfR, following 1h incubation with anti-hTfR mAb at 4°C, cells were incubated with PE-conjugated donkey anti-mouse Ab for 30 min at 4°C. Then, cells were incubated at 37°C for 30 min. Surface-associated Ab was removed by a wash with ice-cold medium 1 for 1 min, followed by two more washes with RPMI1640 plus 10% FBS. The surface-stripped cells were assayed for fluorescence content using flow cytometry. Fluorescence contained in this fraction was designated as the internal fraction. In parallel dishes cells were incubated with mAb and secondary Ab all the time at 4°C. The cells were assayed for fluorescence content which was designated as the surface fraction. The internalization percentage was calculated as a percentage of internal fluorescence intensity to the surface fluorescence intensity.

### Preparation of anti-hTfR mAb conjugated nanoparticles

The HDL-mimetic peptide-lipid nanoparticles (HPPS) which were modified by DSPE-PEG (2000) maleimide around its surface were prepared and purified as reported before [9,10]. 27μl Traut’s reagent (25 mg/ml, Sigma Aldrich, USA) was mixed with 360μl anti-TfR mAb (10 mg/ml) in PBS. The mAb-SH could not be collected until 1h reaction of the mixture under a roller mixer at room temperature. Then a Ultrafiltration device (Amicon Ultra-15, 30000 MWCO, MERK) was used to remove the excess Traut’s reagent. The anti-hTfR mAb conjugated HPPS (HPPS-mAb) was prepared by mixing mAb-SH with maleimide-containing HPPS and shaking at room temperature for 20h. The nanostructure carried DIR-BOA, a near-infrared fluorescent dye as cargo. Concentration of DIR-BOA was determined from a standard curve of DIR-BOA according to the same protocol as described before [10,11].

### hTfR-EGFP targeting specificity of nanoparticles

2×10^5^ CHO-hTfR cells and the same amount of CHO cells were co-incubated with HPPS or HPPS-mAb at concentration of 312.5 nM at 37°C for 30 min. After twice washes, EGFP^+^DIR-BOA^+^ double positive cells were measured by flow cytometry using APC-Cy7 color substitution.

### Statistic analysis

Expression of hTfRs was analyzed by Dunnett-t test and percentage data were analyzed by Mann-Whitney U test using SPSS 17.0 statistical software (SPSS Inc., USA). All values were expressed as means ± SD. Differences were considered to be statistically significant when *P*<0.05.

## Results

### Construction and expression of hTfR-EGFP

**Fig 1:**
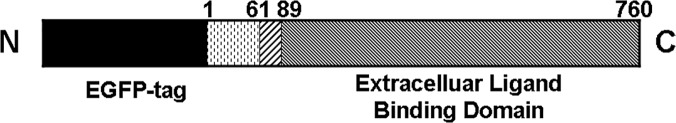
Schematic representation of the hTfR-EGFP chimera. TfR is a type II transmembrane glycoprotein found primarily as a homodimer consisting of identical monomers joined by two disulfide bonds. Each monomer (760 amino acids) consists of three major domains as follows: a large glycosylated extracellular C-terminal domain (amino acids 90–760) involved in ligand binding, a single-pass transmembrane domain (amino acids 62–89), and a short intracellular N-terminal domain (amino acids 1–61). The EGFP moiety is fused to the amino terminus of hTfR.

**Fig 2:**
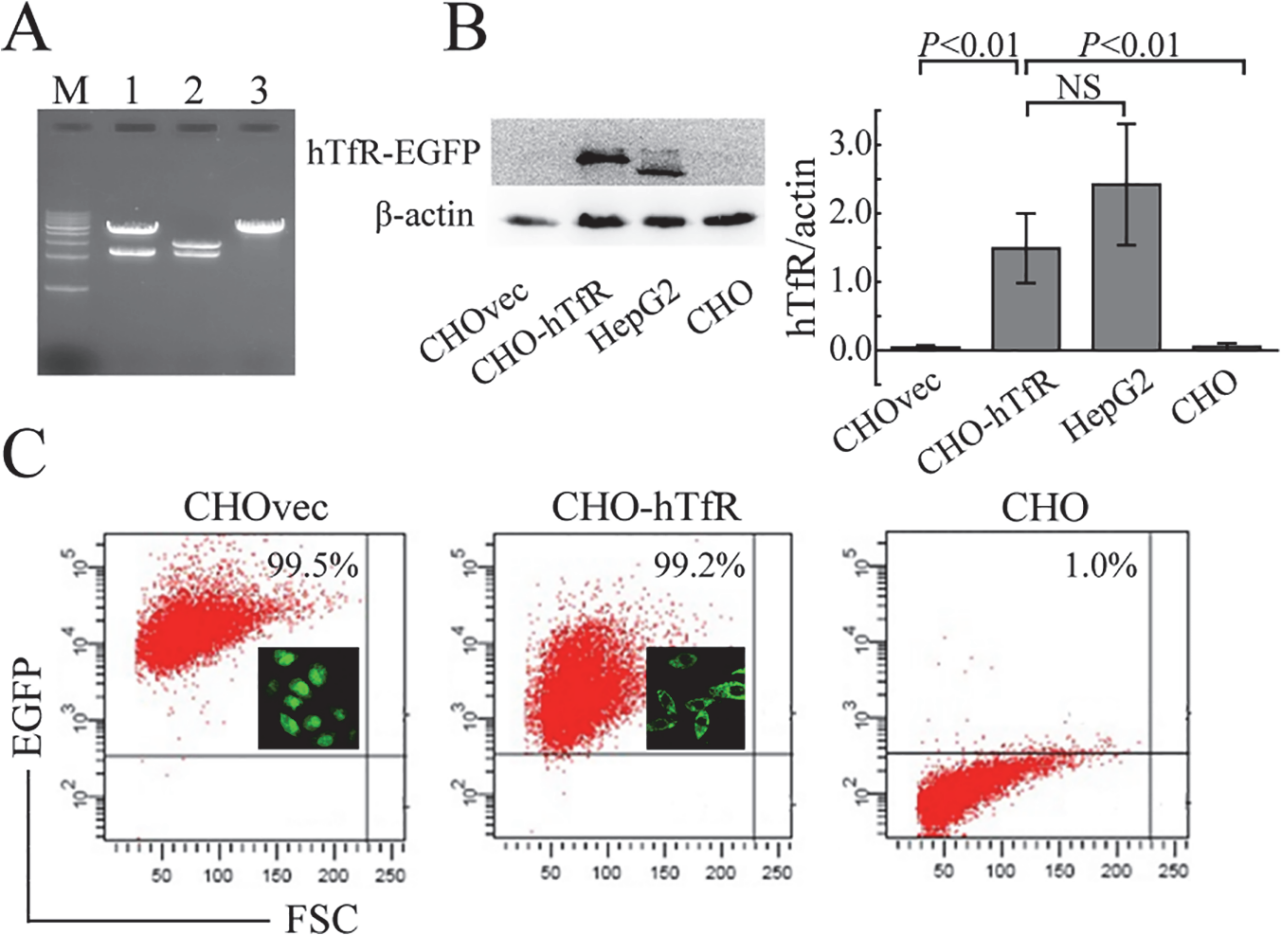
Construction and Expression of hTfR-EGFP in CHO cells. (A) Sal I and BglII restriction enzyme digestion analysis. M: 1kb DNA ladder; lane 1: pEGFP-hTfR, lane 2: pGEM-T-hTfR, lane 3: pEGFP-C1. (B) Immunoblot analysis of hTfR expression in cells. Cell lysates were probed by mouse anti-human TfR mAb. Left: representative WB picture of 4 separate experiments was shown. Right: Densitometric analysis of hTfR levels of the western blots. *P* values were calculated on the basis of Dunnett-t test (NS, not significant). (C) EGFP expression in CHO cells was detected by FCM and fluorescence microscope (insert).

To prepare fluorescent- labeled hTfR, the EGFP was fused to the amino terminus of full-length hTfR (Fig 1). Restriction enzyme digestion (Fig 2A) and DNA sequencing (data not shown) confirmed that *hTfR* cDNA had been successfully cloned into pEGFP-C1 and the predicted amino acid sequence of *hTfR* were in agreement with NM_003234.2 and NP_003225.2 in GenBank database and published reports [12,13].

hTfR-EGFP has been expressed stably in CHO cell lines (Fig 2B). Then cell lysates were probed with antibodies specific for human TfR. Western blot analysis showed a specific band with a molecular weight of about 120kDa emerged in the extract of CHO-hTfR cells but there were no specific bands in the CHOvec and CHO cells. A specific band with molecular weight of about 95kDa emerged in the extract of hTfR-wt HepG2 cells. This anti-hTfR mAb reveals the predicted size for the chimera (120 kDa) and does not identify free hTfR at 95 kDa in CHO-hTfR cells. Furthermore, densitometric analysis suggested that hTfR expression level in CHO-hTfR cells was considerably the same as in HepG2 cells (Fig 2B). Immunofluorescence staining also showed that over 99% stable transfected cells could emit green fluorescence and the chimera was mainly distributed on the membrane of CHO-hTfR cells (Fig 2C). These data confirmed the stability of hTfR-EGFP on cell surface.

### hTfR-EGFP chimera binds with mAb/Tf specifically

**Fig 3:**
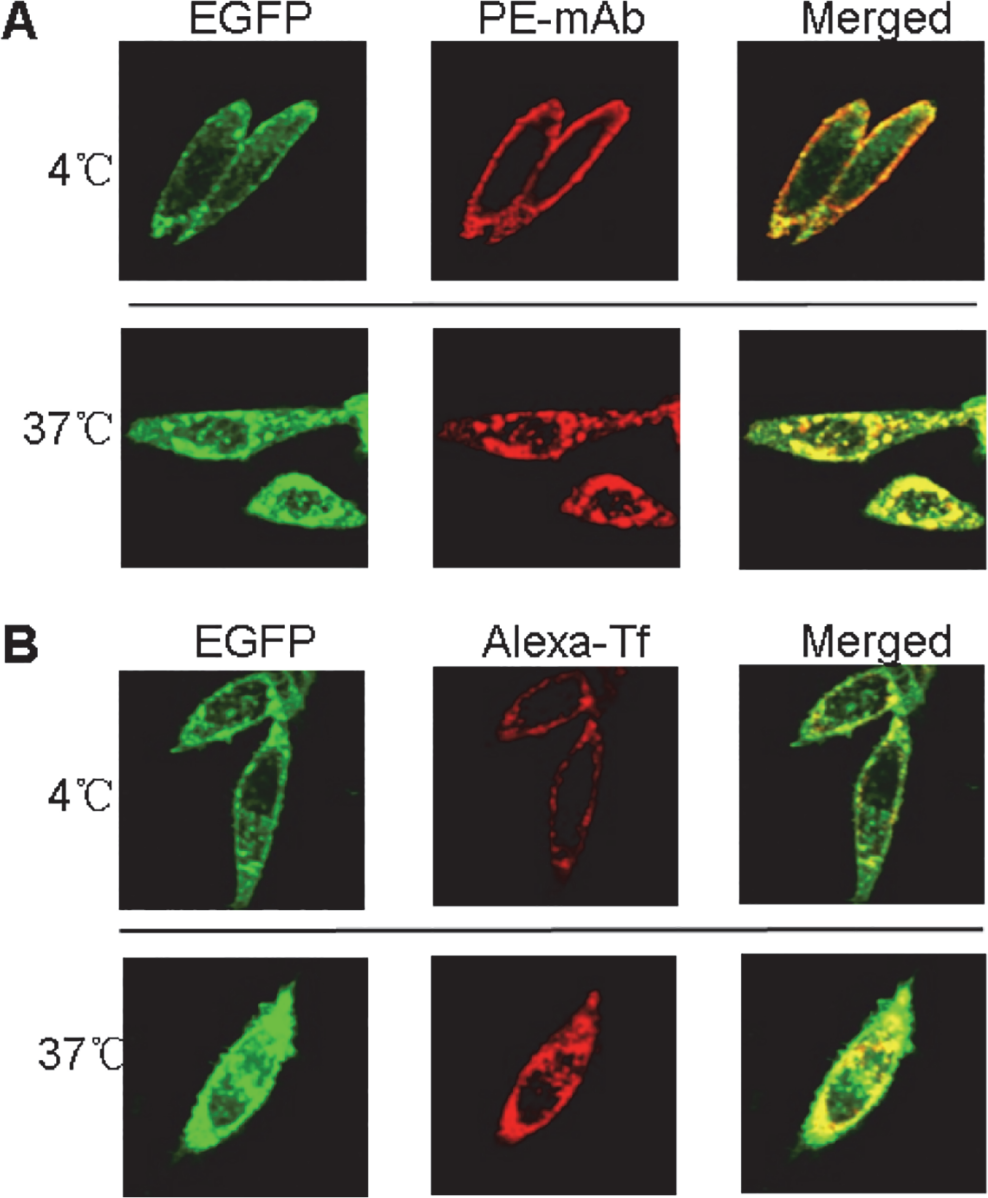
mAb/Tf was bound (4°C) and endocytosed (37°C) by CHO-hTfR cells. (A) 2×10^5^ CHO-hTfR cells were incubated with anti-hTfR mAb for 1h followed by PE-conjugated donkey anti-mouse Ab staining for 30min at 4°C (upper). Half cells were incubated at 37°C for another 30 min and then rinsed by ice-cold medium 1 (Lower). (B) 2×10^5^ CHO-hTfR cells were incubated with Alexa-Tf (5μg per tube) at 4°C (upper) or 37°C for 1h. Then 37°C incubated cells were rinsed with ice-cold medium 1 (lower). Fluorescence was developed using Olympus fluorescence microscope (60×).

To verify the specific binding of hTfR-EGFP chimera with its natural ligand transferrin (Tf) and anti-hTfR mAb, confocal imaging studies of CHO-hTfR cells incubated with mAb or Tf at 4°C and 37°C were conducted. When CHO-hTfR cells were treated with mAb or Tf at 4°C to block vesicular trafficking, the fluorescence of EGFP was visualized mainly at the cell surface. The pattern of staining of the cells with red fluorescence labeled Tf or anti-hTfR mAb labeled by PE-conjugated secondary Ab was identical to that of EGFP. Orange to yellow overlap fluorescence in the merged images suggested the colocalization of hTfR-EGFP and mAb/Tf on the membrane. Moreover, when CHO-hTfR cells were incubated at 37°C for another 30min for endocytosis, the green and red fluorescence still colocalized to redistribute to the periphery and in the perinuclear regions of the cell (Fig 3). Thus, mAb/Tf binding with CHO-hTfR cells was mediated presumably through hTfR. A number of coefficients were calculated to characterize quantitative colocalization (Table 1). The study determined PCC, MOC, overlap coefficients k1 and k2, and colocalization coefficients m1 and m2. PCC indicated numbers which should be interpreted as its presence of colocalization (>0.5) and MOC values were within the range of 0.6–1.0 indicating colocalization. Other coefficients, such as pairs of k1-k2 and m1-m2 also gave us good values indicating colocalization. Therefore, both pairs of mAb-hTfR and TF-hTfR should be considered colocalized.

**Table 1:** Comparison of the results of coefficients calculations.

Coefficient	4°C mAb-hTfR colocalization	37°C mAb-hTfR colocalization	4°C TF-hTfR colocalization	37°C TF-hTfR colocalization

Pearson’s correlation coefficient (Rr)	0.81±0.07	0.90±0.04	0.86±0.03	0.84±0.01
Manders’ overlap coefficient (R)	0.85±0.06	0.92±0.04	0.91±0.03	0.88±0.01
Overlap coefficients k1 and k2	0.83±0.11	1.16±0.04	1.19±0.37	1.24±0.08
	0.87±0.12	0.73±0.08	0.73±0.19	0.62±0.03
Colocalization coefficients m1 and m2	1.00±0.00	1.00±0.00	1.00±0.00	1.00±0.00
	0.88±0.14	0.93±0.06	0.98±0.02	0.93±0.04

Three images from three sections were quantified.

**Fig 4:**
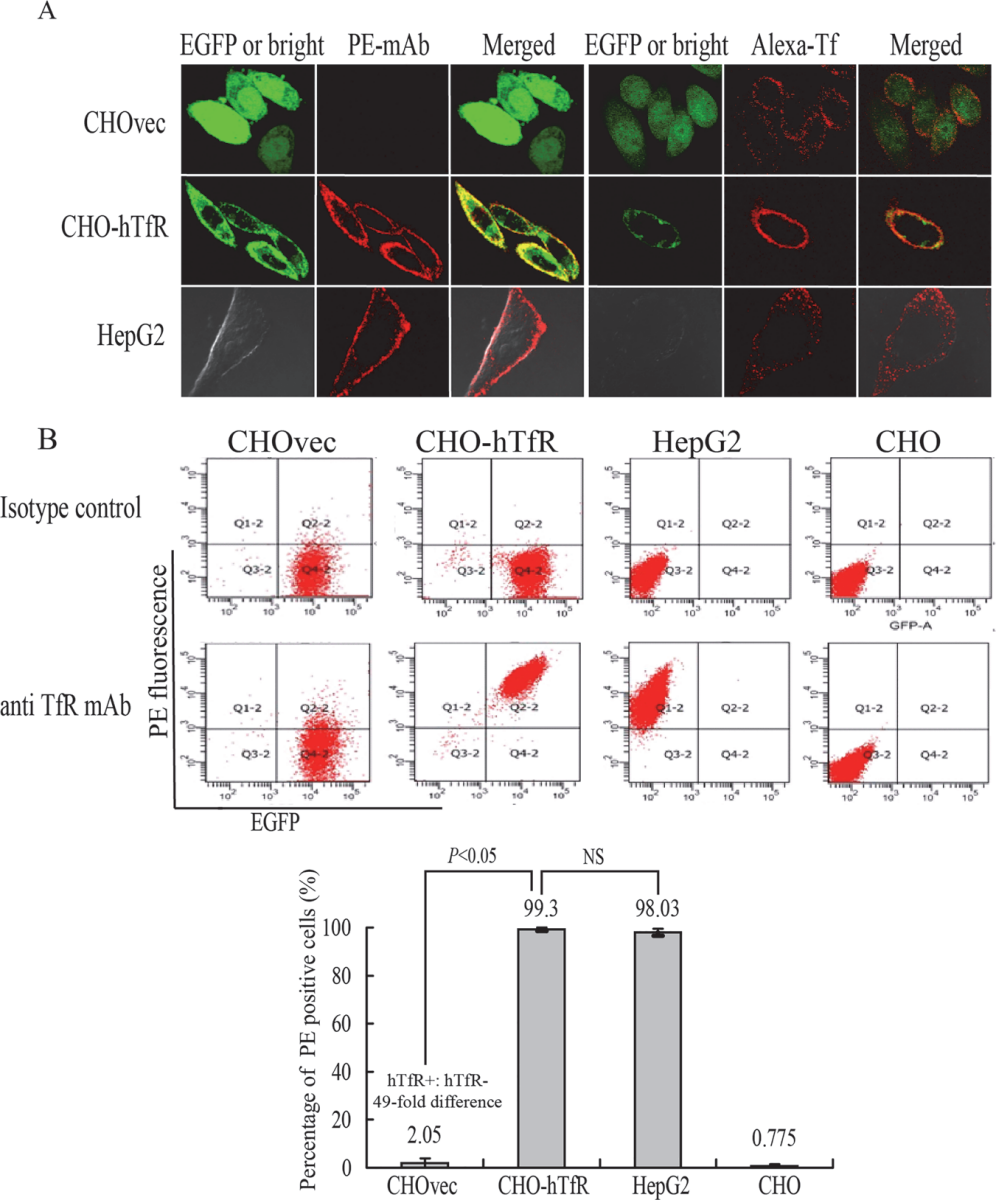
Validation of the hTfR-EGFP specificity. (A) Confocal imaging (4°C) and (B) flow cytometry studies on CHO and CHOvec(hTfR^-^) cells, CHO-hTfR (hTfR^+^) cells, HepG2 (hTfR^+^-wt) cells demonstrated the hTfR targeting of mAb and Tf. (B) Upper: representative FCM pictures were shown. Lower: The bar graph represented FCM analysis of the percentage of PE positive cells. Mean values ± standard deviation (SD), n = 3; *P* values were calculated on the basis of SPSS 17.0 statistical software (NS, not significant).

Next, we examined whether this binding was indeed hTfR-specific. For this purpose, vector control CHOvec cell line was set as a negative control (hTfR^-^) and HepG2 cells as positive control (hTfR^+^) for comparison with results obtained with the CHO-hTfR cells. Confocal imaging studies showed binding of mAb with CHO-hTfR(hTfR^+^) cells and HepG2 cells but not with CHOvec(hTfR^-^) cells (Fig 4A). This result also supported the western blot analysis (Fig 2B) suggesting that no detectable amount of free hTfR was present in hTfR-EGFP expressing cells. HepG2 cells showed a similar distribution of hTfR immunoreactivity, whereas no green fluorescence was detected (Fig 4A), indicating the specificity of the detection of hTfR-EGFP fluorescence in our system.

Although Wei et al [14] showed that human Tf could be internalized by hamster TfR when CHO cells were incubated with Texas red-Tf at 15°C for 2.5h, in our experiment, after incubated with Alexa-Tf at 4°C for 1h, CHOvec cells showed low or undetectable red fluorescence on cell surface. One possible explanation was that under low temperature conditions, human Tf has a much lower affinity for the Chinese hamster TfR than for the human TfR [15,16].

The hTfR-targeting specificity of mAb was further quantified by flow cytometry analysis (Fig 4B). A 49-fold difference (n = 4, *P*<0.05) in mAb binding was observed between CHO-hTfR and CHOvec cells. No difference was observed between CHO-hTfR and HepG2 cells. These results suggested that mAb binding with CHO-hTfR cells was mediated by hTfR. The internalization percentage of this chimera hTfR-EGFP was calculated to be 81±3% (data not shown).

### Endocytosis of hTfR-EGFP in Living CHO-hTfR Cells

**Fig 5:**
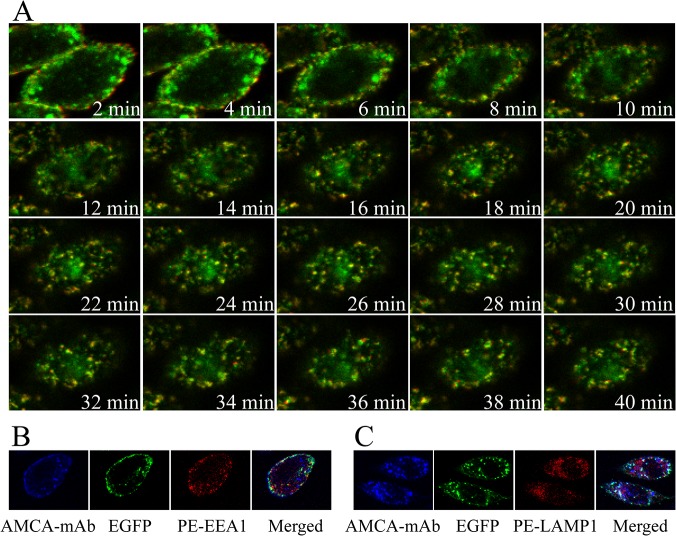
Dynamics of hTfR-EGFP mediated endocytosis in living cells. CHO-hTfR cells expressing hTfR-EGFP were grown in glass chambers overnight. (A) Cells were cultured with anti-hTfR mAb for 1h at 4°C, and then the chamber was mounted onto the microscope stage. 2 min after the supplement of secondary antibody conjugated with PE to the chamber, image acquisition of PE and EGFP fluorescences was performed. Images were acquired continuously at 2-min interval for 40min on a microscope stage. Presented were the selected images taken during this period. Images were indicated by time points. PE and EGFP segregation emerged from 18 min. (B and C) Anti-TfR mAb (AMCA secondary labeling) binding cells were incubated for another 5min or 30min at 37°C to allow internalization. PE conjugated anti- mouse LAMP-1/ EEA-1 antibody was used to label intracellular lysosome and endosome to show the colocalization with mAb and hTfR-EGFP chimera at different time point. EEA-1 for 5min and LAMP-1 for 30min.

The characterization of stable expression of hTfR-EGFP described above suggests that this chimera is indeed an appropriate tool to study hTfR trafficking using optical microscopy of live cells. In following experiments the mAb-induced endocytosis of hTfR-EGFP was visualized in CHO-hTfR cells. An important question that could now be addressed was to compare the post-endocytic localization of the monoclonal antibody, and the hTfR. Cells were allowed to internalize anti-hTfR mAb labeled using PE-conjugated secondary Ab at 37°C, and the time course of cellular distribution of EGFP and PE red fluorescence was monitored in living cells using Olympus imaging system. As shown in Fig 5A, the binding of mAb and its diffuse staining on the cell surface were clearly seen after 2 min of Ab addition. The fluorescent images were acquired following additional 38 min of continuous endocytosis at 37°C. After 6–8 min of incubation, the evenly distributed fluorescence first became ‘grainy’, thereafter, a large part of the grains started to cluster. Green fluorescence was seen to colocalize with internalized red fluorescence and the resulted orange/yellow fluorescence clustered in the same submembrane vesicular structures. The endosomal compartments were rapidly moving and often migrating toward a perinuclear area and had a complex morphology. At the late stages of intracellular trafficking, green and some red fluorescence remained co-localized in the large perinuclear endosomes. However, a small portion of PE fluorescence was associated with the peripheral vesicular compartments that did not contain EGFP (emerged from 18min).

To further confirm the endocytic pathway of the mAb and TfR-EGFP chimera, intracellular localization of TfR-mAb with endosome or lysosome was observed by confocal microscopy in fixed cells. As shown in Fig 5B and 5C, at timepoint 5min, fluorescences of mAb and hTfR were seen to overlap with the fluorescence of EEA-1 which is an early endosome marker. And after 30min, localization of mAb/hTfR complexes and of lysosomal markers (LAMP-1) were visualized.

This real-time fluorescence microscopy of live CHO-TfR cells allows visualization of the dynamics of TfR-EGFP trafficking during the early and later stages of receptor endocytosis that are not preserved in chemically fixed cells.

### CHO-hTfR cells preferentially took up anti-hTfR mAb-conjugated nanoparticles

**Fig 6:**
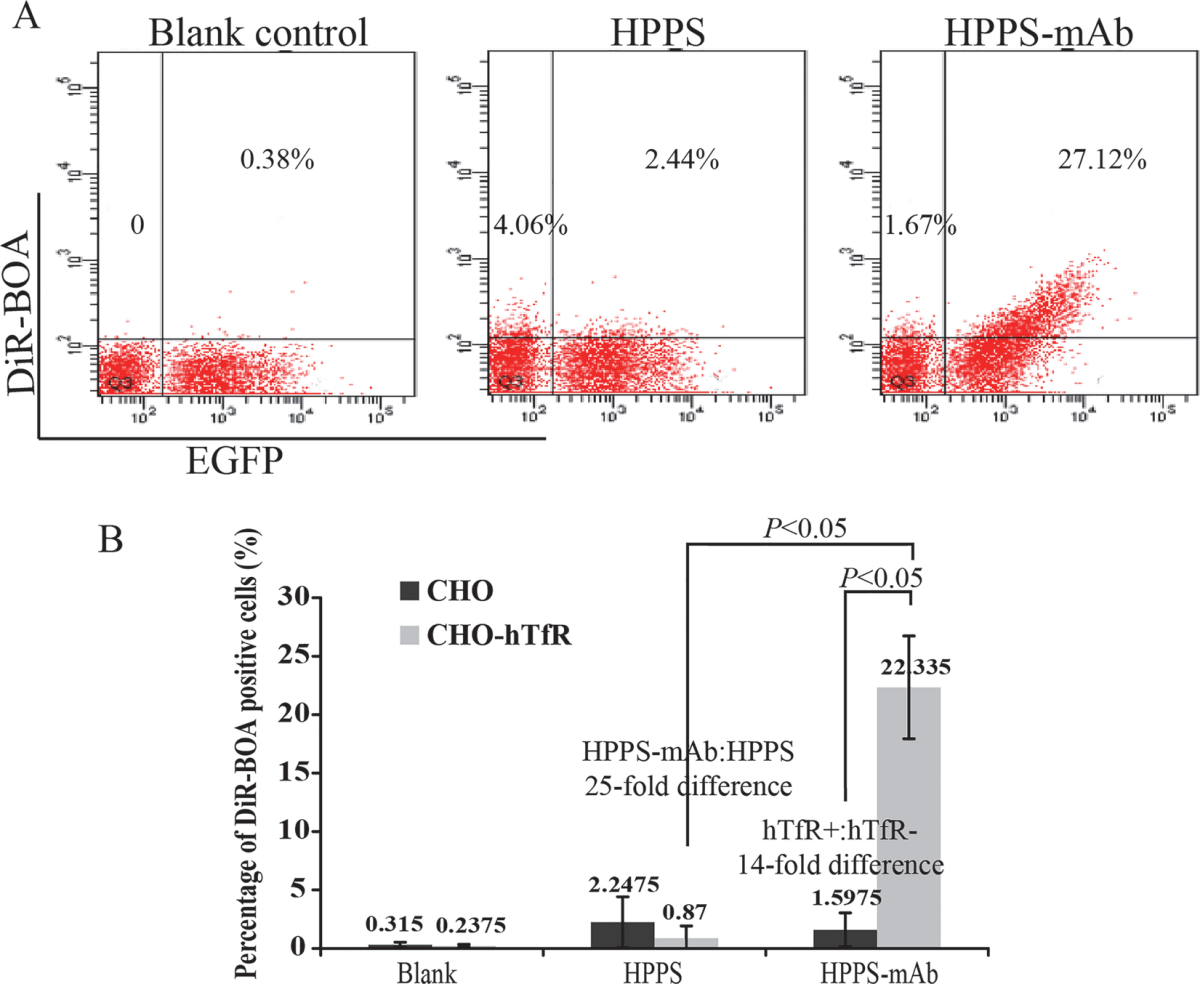
CHO-hTfR cells preferentially took up anti-hTfR mAb-conjugated nanoparticles. The same amounts of CHO-hTfR and CHO cells (2×10^5^) were co-incubated with HPPS or HPPS-mAb at concentration of 312.5 nM at 37°C for 30 min. After twice washes, EGFP^+^DIR-BOA^+^ double positive cells were measured by flow cytometry using APC-Cy7 color substitution. (A) Representative FCM pictures were shown. (B) The bar graph represented FCM analysis of the percentage of DIR-BOA positive cells. Mean values ± standard deviation (SD), n = 4; *P* values were calculated on the basis of SPSS 17.0 statistical software.

To test the potential utility of CHO-hTfR cells for evaluating the hTfR-targeting specificity of cargo delivery, EGFP positive CHO-hTfR cells and the same amount of EGFP negative CHO cells were mixed, then cultured with HPPS nanoparticles carrying a near-infrared fluorescent dye DIR-BOA. As expected, because of the presence of the scavenger receptor class B type I (SR-BI) on CHO cells, EGFP negative cells showed weak uptake of HPPS conjugated with or without anti-hTfR mAb. CHO-hTfR cells also showed a weak uptake of HPPS. However, most of HPPS-mAb could be delivered into these EGFP positive cells, which made them emit the near-infrared fluorescence. And the fluorescent intensity of DIR-BOA in these cells increased as the green fluorescence shifted to the right along the X-axis (Fig 6). That meant the more hTfR-EGFP was expressed on the cell surface the more DIR-BOA could be delivered into cells. A 14-fold difference in DIR-BOA uptake was observed between EGFP^+^ and EGFP^-^ cells and a 25-fold difference between HPPS and HPPS-mAb in CHO-hTfR cells. These data suggested that HPPS-mAb were preferentially uptaken by CHO-hTfR cells and this uptake was hTfR targeting. That mixed culture system was very useful in the evaluation of specificity for hTfR-targeted delivery.

## Discussion

Targeting the TfR has been shown to be effective in delivering therapeutic agents, including chemotherapeutic drugs, toxic proteins, and high molecular weight compounds into cells and causing cytotoxic effects including growth inhibition and/or induction of apoptosis in a variety of malignancies in vitro and in vivo including patients [17–20]. rgoptor mediated cargo transport grains started to cluster. Under physiologic conditions, TfR endocytosis is induced by holo-transferrin (Fe-Tf). TfR capping by anti-TfR antibodies can also induce receptor internalization [6–8,21,22]. Previously, we reported the usage of anti-hTfR scFv fused viral peptide/HLA-A2 complex to redirect cytotoxic T cells of viral specificity to TfR-expressing K562 cells [4] and Tf conjugated polyplexes [5] to deliver therapeutic HIF-1α shRNA into various TfR-expressing tumor cell lines. And now we are developing several other Ab conjugated nanoparticles for biomedical applications. In testing the efficacy of this TfR directed cargo delivery into human cancer cells, a negative control cell line which expresses no hTfR on cell membrane is needed for comparison with results obtained from hTfR^+^ cells to assure this hTfR-targeting specificity. Little or no hTfR expression has been only detected on mature erythroid cells and pluripotent hematopoietic stem cells [23]. TfR is essential for highly proliferating malignant cells in iron uptake and hTfR knockdown may affect the regulation of cell growth [24], so knocking hTfR down in any cancer cells to achieve such negative control cell line is not an appropriate option. In this study, CHO cell line expressing no hTfR was modified to express hTfR and then used for comparison with results obtained from hTfR^-^ CHO cells to assure the hTfR-targeting specificity.

For real-time optical analysis of protein trafficking in individual cells, EGFP moiety was fused to the hTfR. TfR is a type II transmembrane glycoprotein contains a large extracellular C-terminal domain involved in ligand binding, a transmembrane domain and a short intracellular N-terminal domain [2,23]. The fusion of EGFP to the amino terminus of TfR could be expected to interfere with processing of newly synthesized TfR but placement of the EGFP at the carboxyl terminus would possibly hinder the ligand binding. Considering these, the GFP moiety was attached to the amino terminus of the receptor. Characterization of the hTfR-EGFP chimera showed that it behaved essentially unperturbed in its cellular and biochemical properties when compared with hTfR-wt. hTfR-EGFP mediated mAb and Tf endocytosis was normally judged by confocal images. Apparently, the attachment of EGFP did not constrain synthesis, distribution and function of hTfR.

We took advantage of the GFP fluorescence to simultaneously follow the localization of both hTfR and mAb in living cell. It was found that hTfR-EGFP and mAb labeled using secondary antibodies were mainly localized in the same compartments during endocytosis and the co-localization was prolonged when mAb was continuously present. But a small part of PE fluorescence and green fluorescence became segregated at later stages (emerged from 18min). It was reported that TfR recycling is completed within 15 min [25], so it was possible that mAb dissociated at the late stages of endocytosis from the hTfR-EGFP to be accumulated in vesicular structures and hTfR-EGFP was recycled back or on the way to the cell surface. These observations were not consistent with the hypothesis that the bulk of TfR-Tf complexes remain intact in acidic endosomes and finally are recycled back to the cell surface where Tf is then released [2,23]. Lepelletier Y *et al* [21,26] reported that their anti-hTfR mAb A24 could impair TfR recycling through receptor degradation, leading to a reduced Fe-Tf uptake. However, it is not clear whether our mAb also induces TfR endocytosis in such a nonphysiologic setting. This possibility requires further investigation. Another possibility might be that EGFP faded faster than phycoerythin or displayed quenching [27] within acidic endosomes.

The internalization percentage of hTfR-EGFP chimera was about 81±3% of wild type. This figure is essentially similar to that of native hTfR reported by van Renswoude *et al* [28]. They used another hTfR expressing cell line K562 to be incubated with FITC-Tf and ^125^I-Tf and found only 10–15% of the cell-associated Tf could be removed by acid stripping. Thus, hTfR-EGFP might be useful for biochemical analyses of the receptor function.

Western blot analysis demonstrated no free hTfR present in the extract of CHO-hTfR cells. This result suggested the mAb produced by our lab only showed specificity to human TfR and showed no cross reactivity to hamster TfR. This will make it more credible for our data obtained from following *in vivo* tumor-bearing mice experiments when our mAb or mAb derivatives conjugated nanoparticles are applied in cancer therapy.

In summary, the present study was undertaken to address the establishment of CHO-hTfR cell line which stably expressing hTfR fused with EGFP. We provide evidence here that this cell line retains endocytosis function as hTfR-wt cells and makes it feasible for accurate evaluation and visualization of intracellular trafficking of therapeutic agents conjugated with transferrin or Abs targeting the hTfRs.
